# Comparison of the NIST and BIPM Standards for Air Kerma in Medium-Energy X-Rays

**DOI:** 10.6028/jres.111.028

**Published:** 2006-10-01

**Authors:** D. T. Burns, M. O’Brien

**Affiliations:** Bureau International des Poids et Mesures, F-92312 Sèvres Cedex, France; National Institute of Standards and Technology, Gaithersburg, MD 20899 USA

**Keywords:** air kerma, free-air ionization chamber, primary standard, reference radiation qualities, medium-energy x-rays, x-ray calibration

## Abstract

A comparison has been made of the air-kerma standards for medium-energy x-rays of the National Institute of Standards and Technology (NIST) and the Bureau International des Poids et Mesures (BIPM). The comparison involved a series of measurements at the BIPM and the NIST using the air-kerma standards and three NIST reference-class transfer ionization chamber standards. Reference beam qualities in the range from 100 kV to 250 kV were used. The results show the standards to be in reasonable agreement within the combined standard uncertainty of the comparison of 0.37 %, although a significant trend with radiation quality is observed and the possible sources discussed.

## 1. Introduction

An indirect comparison was made between the air-kerma primary standards for medium-energy x-rays of the National Institute of Standards and Technology (NIST) and the Bureau International des Poids et Mesures (BIPM). The measurements were conducted at both laboratories between November 2002 and June 2003 using the tungsten reference radiation qualities in the range from 100 kV to 250 kV recommended by the Consultative Committee for Ionizing Radiation (CCEMRI) [1]. Three NIST reference-class ionization chambers were used as transfer instruments for the comparison.

## 2. Determination of the Air-Kerma Rate

For a free-air ionization chamber standard with measuring volume *V*, the air-kerma rate is determined by the relation

$$\dot{K}=\frac{I}{\rho_{\text{air}}V}\frac{W_{\text{air}}}{e}\frac{1}{1-g_{\text{air}}}\prod_{i}k_{i},$$ (1)

where

*I*/*ρ*_air_*V* is the mass ionization current measured by the standard,
*W*_air_ is the mean energy expended by an electron of charge *e* to produce an ion pair in dry air,
*g*_air_ is the fraction of the initial electron energy lost by bremsstrahlung production in air, and
Π *k*_i_ is the product of the correction factors to be applied to the standard.

The values for the physical constants used in the determination of the air-kerma rate are given in Table 1.

## 3. Characteristics of Chambers

### 3.1 Description of Air-Kerma Standards

Both air-kerma standard chambers used in the comparison are parallel-plate free-air ionization chambers. The measuring volume *V* is defined by the diameter of the chamber aperture and the length of the collecting plate. The BIPM air-kerma standard is described in Refs. [2] and [3]. The NIST Wyckoff-Attix chamber is described in Refs. [4] and [5]. The main dimensions, the measuring volume and the polarizing voltage for each chamber are given in Table 2.

### 3.2 Description of Transfer Ionization Chambers

Three NIST transfer chambers (NIST-T1, NIST-T2, NIST-T3, serial numbers 2022, 2023, and 260 respectively) were used for the comparison. Each was calibrated at the NIST before and after a series of calibrations at the BIPM in February 2003. All three transfer standards are spherical and constructed with a wall of air-equivalent plastic. Each has a nominal volume of 3.6 cm^3^, an external diameter of 1.9 cm and a wall thickness of 0.25 mm. A collecting voltage of 300 V was applied to each standard. Measurements were made using both polarities at each laboratory, although the comparison results are derived from the negative polarity measurements only.

## 4. Comparison Details

### 4.1 Irradiation Facilities and Reference Beam Qualities

The BIPM measurements were made at the BIPM medium-energy x-ray laboratory which, at the time of the comparison, housed a constant-potential generator (maximum usable generating potential 250 kV) and a tungsten-anode x-ray tube with an inherent filtration of around 2.3 mm of aluminum. Both the generating potential and the tube current were stabilized using feedback systems constructed at the BIPM, resulting in high stability and eliminating the need for a transmission current monitor. For a well-behaved transfer instrument, the variation in the measured ionization current over the duration of a comparison was typically less than 0.04 %. The BIPM radiation qualities in the energy between 100 kV and 250 kV are those recommended by CCEMRI [1] and are given in Table 3.

The NIST measurements were made using the Wyckoff-Attix free-air chamber in the NIST 300 kV calibration facility. The x-ray source at the time of the comparison was a 320 kV x-ray generator with a metal-ceramic x-ray tube, both supplied by Seifert-Pantak^1^. The x-ray generator is a high-frequency, highly stabilized voltage source. The tungsten anode x-ray tube, model MXR-321, has a beryllium window of thickness 3 mm and a focal spot 8.0 mm in diameter. The materials used for the filtration and for the measurement of HVL were at least 99.99 % pure with thicknesses known with an uncertainty of 0.01 mm. The output stability was monitored using a transmission ionization chamber. The reference radiation qualities used at the NIST were generated between 100 kV to 250 kV and are listed in Table 3.

### 4.2 Correction Factors

Although free-air chambers are designed to minimize or eliminate corrections to the measured ionization current, certain corrections are unavoidable. The correction factors applied to each free-air chamber and the associated uncertainties are listed in Tables 4 and 5.

A correction must be made for the attenuation of the x-ray fluence along the air path between the reference plane and the center of the collecting volume. The correction factor *k*_a_ is calculated using the measured air-attenuation coefficients *µ*_air_, according to

$$k_{a}=\exp(\mu_{\text{air}}L)$$ (2)

The effective attenuation path L varies with the temperature and pressure of the air in the chamber and so the values for *k*_a_ are corrected for this effect. All ionization measurements are also corrected for the temperature and pressure of the ambient air between the radiation source and the reference plane.

All measured ionization currents using the free-air chamber standards are corrected for ion recombination, *k*_s_. The ionization currents measured with the transfer standards are not corrected for ion recombination. However, since the air-kerma rates used at both facilities are low, minimal volume recombination occurs in the transfer chambers. This was demonstrated by making separate recombination measurement at the BIPM for two of the three transfer chambers. From these one can conclude that the different air-kerma rates at the NIST and the BIPM do not affect the comparison results by more than 0.02 %. The standard chambers are corrected for the humidity effect *k_h_* which is taken as 0.998 for both standards and all radiation qualities.

### 4.3 Chamber Positioning and Measurement Procedure

Each calibration was made by alternating between the transfer chamber and the standard free-air chamber, this being achieved by movement of the x-ray tube at the BIPM and by translation of the chambers at the NIST. At both laboratories alignment on the beam axis was measured to around 0.1 mm and this position was reproducible to better than 0.01 mm, as observed by an alignment telescope. No correction is applied for the radial non-uniformity of each beam. The reference plane for each chamber was positioned at 1000 mm from the radiation source at the NIST and 1200 mm at the BIPM. The beam diameter in the reference plane was 60 mm at the NIST and 83 mm at the BIPM.

The leakage current was measured before and after each series of ionization current measurements and a correction made based on the mean of these leakage measurements. For all measurements the leakage current was less than 0.01 % of the ionization current. For all chambers at the NIST a total of not less than 30 measurements with an integration time of 60 s were made. The statistical standard uncertainty of the current measurements was typically less than 0.1 % for the NIST transfer chambers. At the BIPM each calibration coefficient was obtained from a total of 14 measurements of around 60 s performed with the BIPM standard and each transfer chamber. The statistical standard uncertainty of each calibration coefficient is less than 0.02 %. Repeatability of the calibration coefficient on different days, after having removed and replaced the chamber, was typically 0.03 %.

The polarity correction for the transfer chambers was measured at the BIPM to be only typically 1.0006 for all transfer chambers. Only the negative polarity measurements are used for the comparison results.

All transfer ionization chamber current measurements were normalized to 293.15 K and 101 325 Pa. The transfer chamber currents are not corrected for humidity. The humidity is monitored and recorded at both facilities. The BIPM laboratory humidity is maintained at 50 %. The NIST laboratory average humidity is 30 %.

## 5. Measurement Uncertainties

The uncertainties associated with the primary standards are given in Table 6. The NIST uncertainties were evaluated according to Ref. [6]. The uncertainties associated with the calibration of the transfer ionization chambers at the NIST and at the BIPM are listed in Table 7. The type A uncertainty of the ionization current includes a component for the reproducibility of calibrations at each laboratory. For the BIPM, this is 0.03 %, based on repeat calibrations of the NIST transfer chambers on different days. For the NIST, the value 0.12 % is derived from the difference in the mean calibration coefficients before and after the measurements at the BIPM. The uncertainty of the comparison results is given in Table 8.

## 6. Results and Discussion

The results for the transfer ionization chamber calibrations at both laboratories are shown in Table 9. For each transfer chamber and each radiation quality, the ratio of the calibration coefficients *N_K_*,_NIST_/*N_K_*_,BIPM_ is evaluated, as shown in Table 10. Also given in the table is the standard uncertainty of the distribution for each set of three determinations. This does not represent the comparison uncertainty, but rather the consistency of the results determined with the different transfer chambers. There is no clear reason for the higher value of 0.29 % at 250 kV, but the results at the other three qualities are consistent with the values determined for the reproducibility of NIST calibrations (0.12 %) noted in the preceding section.

The comparison result *R_K_* for each radiation quality is the mean of the three ratios for that quality. These are given in Table 11 and show evidence of a trend with beam quality, decreasing by 0.8 % between 100 kV and 250 kV. This trend has been seen previously in certain comparisons at the BIPM and no reason has been found. One possibility under study is the dependence of the calibration coefficients on field size; the field sizes at the NIST (60 mm) and at the BIPM (83 mm) are quite different.

The comparison uncertainty given in Table 11 takes into account correlations in the BIPM and NIST values for the physical constants and the humidity correction. Correlations also exist in the values for *k*_e_, *k*_sc_, and *k*_fl_, since both laboratories use values derived from the same Monte Carlo calculations [7]. These are accounted for by taking half the uncertainty value for each component at each laboratory, in accordance with the analysis made for the degrees of equivalence appearing in the BIPM key comparison database. All other correction factors are assumed to be uncorrelated.

Comparing these results with those of the NIST/BIPM comparison of 1991, there is reasonable consistency within the uncertainties, except for a difference exceeding 0.5 % at 100 kV. Although a number of corrections factors, for example *k*_e_ and *k*_sc_, have changed, and the factor *k*_fl_ has been introduced at both laboratories, the most notable change is in the air-attenuation correction at the NIST. For 100 kV, this factor was 1.0097 in 1991 (HVL = 5 mm Al) and is now 1.0152 (HVL = 3.943 mm Al), a change that accounts for most of the difference seen in the comparison results at 100 kV. For comparison, the BIPM value is 1.0102 (HVL = 4.027 mm Al), which is much closer to the NIST value from 1991 than it is to the current NIST determination.

These results will be used as the basis of degrees of equivalence for the NIST medium-energy x-ray standard in the BIPM key comparison database.

## Figures and Tables

**Table 1:** Physical constants used in the determination of the air-kerma rate

Physical constant	Value	Relative standard uncertainty (%)
*ρ*_air_^a^	1.293 kg m^−3^	0.01
*W*_air_/*e*	33.97 J C^−1^	0.15

a Density of dry air at 273.15 K and 101 325 Pa.

**Table 2:** Main characteristics of the primary standards used in the comparison

Characteristic	NIST	BIPM
Air-path length/cm	30.8	28.15
Plate separation/cm	20.0	18.0
Collecting plate length/cm	10.08	6.0004
Aperture diameter/cm	0.9999	0.9939
Measuring volume/cm^3^	7.915	4.6554
Polarizing voltage/V	−5000	4000

**Table 3:** Characteristics of the reference radiation qualities used for the comparison

Reference radiation	Generating potential kV	Additional filtration		Half-value layer (HVL)		Attenuation coeff *µ*_air_^a^ m^−1^	Air-kerma rate mGy s^−1^
		mm Al	mm Cu	mm Al	mm Cu		
BIPM							
100 kV	100	1.2032		4.027	0.148	0.0355	0.21
135 kV	135		0.2321		0.494	0.0235	0.21
180 kV	180		0.4847		0.990	0.0198	0.30
250 kV	250		1.5701		2.500	0.0172	0.39
NIST							
100 kV	100	3.248		3.943	0.149	0.0493	1.04
135 kV	135	1.060	0.265		0.496	0.0270	1.00
180 kV	180	3.842	0.482		1.003	0.0257	1.30
250 kV	250	3.842	1.618		2.502	0.0179	1.58

a Air attenuation coefficient at 293.15 K and 101 325 Pa, measured at the BIPM for an air path length of 270 mm and at the NIST for 308 mm.

**Table 4:** Correction factors used in the comparison for the NIST Wyckoff-Attix standard

Correction factor	Generating potential (kV)				Relative standard uncertainty (%)	
	100	135	180	250	Type A	Type B
Air attenuation *k*_a_^a^	1.0152	1.0083	1.0079	1.0055	0.12	0.02
Scattered radiation *k*_sc_	0.9942	0.9952	0.9958	0.9969	–	0.07
Electron loss *k*_e_	1.0000	1.0006	1.0027	1.0055	–	0.05
Ion recombination *k*_s_	1.0004	1.0001	1.0006	1.0002	0.1	–
Fluorescence k_fl_	0.9981	0.9991	0.9995	0.9999	–	0.03
Aperture transmission *k*_l_^b^	1.0000	1.0000	1.0000	1.0000	–	0.04
Field distortion *k*_d_	1.0015	1.0015	1.0015	1.0015	–	0.2
Polarity effect *k*_pol_	1.001	1.000	1.001	1.001	0.05	–
Wall transmission *k*_p_^b^	1.0000	1.0000	1.0000	1.0000	–	0.01
Bremsstrahlung 1 − *g*_air_	1.0000	1.0000	1.0000	1.0000	–	0.01
Humidity *k_h_*	0.998	0.998	0.998	0.998	–	0.03

a These are nominal values for *T* = 293.15 K and *p* = 101 325 Pa. Each measurement is corrected using the air temperature and pressure measured at the time.

b The uncertainties in the aperture and wall transmission corrections are negligible.

**Table 5:** Correction factors used in the comparison for the NIST Wyckoff-Attix standard

Correction factor	Generating potential (kV)				Relative standard uncertainty (%)	
	100	135	180	250	Type A	Type B
Air attenuation *k*_a_^a^	1.0102	1.0067	1.0057	1.0049	0.03	0.01
Scattered radiation *k*_sc_^b^	0.9952	0.9959	0.9964	0.9974	–	0.03
Fluorescence *k*_fl_^b^	0.9985	0.9992	0.9994	0.9999	–	0.03
Electron loss *k*_e_^b^	1.0000	1.0016	1.0043	1.0073	–	0.09
Ion recombination *k*_s_	1.0004	1.0005	1.0005	1.0003	0.02	0.01
Field distortion *k*_d_	1.0000	1.0000	1.0000	1.0000	–	0.07
Aperture transmission *k*_1_	0.9999	0.9998	0.9997	0.9996	–	0.01
Wall transmission *k*_p_	1.0000	0.9999	0.9999	0.9988	0.01	–
Bremsstrahlung 1 − *g*_air_	0.9999	0.9999	0.9998	0.9997	–	0.01
Humidity *k_h_*	0.998	0.998	0.998	0.998	–	0.03

a These are nominal values for *T* = 293.15 K and *p* = 101 325 Pa. Each measurement is corrected using the air temperature and pressure measured at the time.

b Values for *k*_sc_, *k*_fl_, and *k*_e_ adopted in October 2003, based primarily on Monte Carlo calculations.

**Table 6:** Relative standard uncertainties (in %) associated with the standards

Source of uncertainty	NIST		BIPM	
	Type A	Type B	Type A	Type B
Ionization current	0.15	0.06	0.03	0.02
Volume	0.04	0.01	0.01	0.05
Positioning	–	0.01	0.01	0.01
Correction factors (excl. *k*_h_)	0.16	0.22	0.04	0.12
Humidity *k*_h_	–	0.03	–	0.03
Physical constants	–	0.15	–	0.15
${\dot{K}}_{{}_{\text{LAB}}}$	0.23	0.27	0.05	0.20

**Table 7:** Relative standard uncertainties (in %) associated with the calibration of the transfer ionization chambers

Source of uncertainty	NIST		BIPM	
	Type A	Type B	Type A	Type B
Air-kerma rate ${\dot{K}}_{{}_{\text{LAB}}}$	0.23	0.27	0.05	0.20
Ionization current	0.12	0.06	0.03	0.02
Positioning	–	0.01	0.01	0.01
*N_K_*_,LAB_	0.26	0.28	0.06	0.20

**Table 8:** Relative standard uncertainties (in %) associated with the comparison results *R_K_*

Source of uncertainty	Type A		Type B
	0.27		0.25
Comparison result *R_K_*			
		0.37	

**Table 9:** Measured results for the calibration of the NIST transfer chambers at both laboratories

Reference radiation	Half-value layer (HVL)		NIST transfer chambers Calibration coefficients (10^6^ Gy C^−1^)		
	mm A1	mm Cu	NIST-T1	NIST-T2	NIST-T3
BIPM					
100 kV	4.027	0.148	8.382	8.469	7.965
135 kV	–	0.494	8.477	8.539	8.080
180 kV	–	0.990	8.608	8.658	8.182
250 kV	–	2.500	8.768	8.813	8.290
NIST					
100 kV	3.943	0.149	8.388	8.465	7.983
135 kV	–	0.496	8.440	8.492	8.056
180 kV	–	1.003	8.576	8.619	8.164
250 kV	–	2.502	8.704	8.727	8.257

**Table 10:** Results of the comparison for each of the transfer chambers

Reference radiation	*^N^K*,NIST^/^*^N^K*,BIPM			Mean for the transfer chambers	
	NIST-T1	NIST-T2	NIST-T3	mean *^N^K*,NIST^/^*^N^K*,BIPM	Std unc of distribution
100 kV	1.0008	0.9995	1.0022	1.0008	0.14 %
135 kV	0.9956	0.9944	0.9970	0.9957	0.13 %
180 kV	0.9962	0.9954	0.9978	0.9965	0.12 %
250 kV	0.9927	0.9903	0.9961	0.9930	0.29 %

**Table 11:** Results *R_K_* of the comparison of the NIST and BIPM air-kerma standards

Reference radiation	*R_K_* (2003)	Standard uncertainty (%)	*R_K_* (1991)
100 kV	1.0008	0.37	0.9952
135 kV	0.9957	0.37	0.9953
180 kV	0.9965	0.37	0.9942
250 kV	0.9930	0.37	0.9930

## Footnotes

1 Certain commercial equipment, instruments, or materials are identified in this paper to foster understanding. Such identification does not imply recommendation or endorsement, nor does it imply that the equipment identified are necessarily the best available for the purpose.
